# Evaluation of saline and magnetized water on emitter hydraulic performance and clogging in drip irrigation

**DOI:** 10.1038/s41598-024-57543-8

**Published:** 2024-03-28

**Authors:** Heba Abdelsalam, Harby Mostafa, Mohamed El-Ansary, Montaser Awad, Wael Sultan

**Affiliations:** 1 Agric. and Biosys. Eng. Dept., Faculty of Agriculture, Benha University, Moshtohor, Qalyobia Egypt; 2 Agric. Eng. Institute, Agricultural Research Center, Giza, Egypt

**Keywords:** Magnetization, Salinity, Hydraulic parameters, Drip irrigation, Drought, Salt

## Abstract

The present investigation was carried out at the National Irrigation Laboratory of Agricultural Engineering Research Institute (AEnRI), Dokki, Giza, Egypt. This study was performed to investigate the hydraulic performance and clogging ratio of drip irrigation with magnetized water. Magnetized water was created by transferring water through a permanent magnet connected to a feed pipeline. Two main treatments of magnetized and non-magnetized water, as well as three sub-treatments of irrigation water salts, including fresh water (219 ppm) and the addition of 1000 and 2000 ppm to irrigation water with three replications were applied under different operating pressure (75, 100, 125 and 150 kPa). At the beginning of the experiment, results show that hydraulic parameters were almost the same for both the magnetized and the non-magnetized water and for all salinity levels. At the end of working time, the hydraulic parameters were improved for the magnetized water under salinity levels compared to the non-magnetized water. Average emitter discharge increased with roughly 2.7% and 5.6%, coefficient of variation (Cv) decreased by 0.6 and 0.91%, emission uniformity (Eu) increased about 1 and 1.1% and variation of average flow rate (q_avr_) decreased by 21.3 and 29.4% when 1000 and 2000 ppm were used, respectively. Magnetized water had slight effect on clogging at non-saline water at the end of experiment. At 1000 and 2000 ppm salinity levels, the clogging ratio decreased by 1.97 and 2.45% at different pressure, respectively. The results show that magnetized water treatment could effectively relieve and delay the occurrence time of clogging.

## Introduction

The primary goal of agriculture is to utilize both available and unusual water due to the scarcity of water resources. Wars over freshwater resources have increased as a result of rising population, more profitable activity, and improved standards. As a result, in order to maintain the current rate of population growth, more agricultural land must be provided to increase food production. These factors make it crucial to employ recycled water, low salinity water, and medium salinity water for irrigation, as well as making greater use of water resources that are currently available. One of the primary issues in agriculture is the usage of excessive salinity, low quality irrigation water. Magnetized water can be used to recover soil and water while reducing soil moisture stress^1–3^.

Drip irrigation is one of the new irrigation techniques that is rapidly gaining popularity. With careful management, drip irrigation may achieve water distribution consistency of up to 95%. Small emitters that are buried or set on the soil's surface and release water at a regulated rate make up drip irrigation systems. Frequent water application helps to maintain appropriate soil moisture conditions, hence reducing moisture stress in the plant^4^. Drip irrigation systems require continual maintenance in such circumstances. The main difficulty and concern with these systems is emitter clogging, which has a negative influence on water distribution uniformity.

Emitter clogging is a phenomenon whereby solid particles, chemical precipitation, microorganisms, and other substances in irrigation water deposit in the lateral or emitters of drip irrigation, resulting in a decrease in irrigation flow rate and uniformity^5,6^. It is a complicated and unavoidable process in agricultural practices^7^. Generally, according to the recommendations given by the International Organization for Standardization, the standard for determining clogging is defined as the actual flow rate of the emitter less than 75% of the design^8^. According to the statistics of a consultant from the Food and Agriculture Organization, the probability of physical, chemical, and biological clogging in drip irrigation accounts for 31%, 22%, and 37%, respectively, while the other types account for 10%. Physical clogging is caused by organic or inorganic suspended matter, such as algae, phytoplankton, and zooplankton residues, plastic fragments, sand, silt, and clay particles that cannot be filtered out in irrigation water using filtration equipment^9^. Chemical clogging is caused by the soluble substances in water sources, such as carbonates, phosphates, sulfates, silicates, hydroxides, Fe^2+^, Ca^2+^, Mg^2+^, and sulfides, that form chemical precipitates under certain conditions^10^. The use of groundwater, saline water, and fertigation for irrigation can cause chemical clogging. Chemical precipitation, a common method of controlling clogging, can be greatly avoided by lowering the pH of irrigation water with acidic chemicals. However, it has significant operator needs and runs the danger of crop damage, soil acidification, and etching of the drip irrigation system^6,10^. Therefore, it is imperative to create a more ecologically friendly, safe, and effective emitter clogging control technique.

According to Aali et al.^11^, magnetization technology was first developed for the industrial descaling of boilers and heat exchangers. The larger cluster structure of water molecules shrinks when it passes through a magnetic field, altering the water's permeability, ionic hydration reaction, and solubility enhancement^12^. This increases the solution's solubility to scale substances in order to achieve physical descaling^13^. The application of magnetization technology to several domains, including agriculture, has been progressively expanding. Magnetized water irrigation has been demonstrated in studies to improve saline land^14^, maintain soil moisture^15^, and maximize yield^16,17^. To address the issue of chemical clogging of emitters, several researchers have creatively added magnetism to drip irrigation systems using brackish water in recent years. Irrigation water quality influences the magnetization effect and that magnetization treatment can reduce clogging in brackish water drip irrigation systems^18,19^.

Sahin et al.^18^ assessed the clogging of emitters with magnetized saline water. They discovered that while using magnetized water, emitters discharged more than when using non-magnetized water. According to Shaker et al.^20^, emitters discharged 3.75 and 3.46 Lh^−1^ in magnetized and non-magnetized water treatments, respectively. The results of Nikbakht et al.^21^ found that using magnetic water, as opposed to the absence of magnetic water, enhanced the hydraulic parameters for assessing the tape drip irrigation system. When compared to no-magnetic water, magnetic water enhanced the system discharge's mean by 4.2% and reduced the coefficient of variation by 0.98%. It also lessens the amount of calcite deposits that chemically clog emitter nozzles. Shi et al.^22^ found that, as compared to non-magnetized water treatments, magnetized water treatment could successfully relieve emitter clogging and delay the occurrence time of clogging, increasing the average discharge variation rate by 37–61.64%. The application of magnetic treatment to saline water resulted in a large 51.3% increase in crop growth rate^23^. Additionally, the treatment reduced the original value of soil salt by 35%, whereas untreated saline water caused an increase of 3.7%. Statistical uniformity coefficients, which indicate emitter clogging in irrigation systems, were found to be 75% for magnetically treated saline water and 48% for untreated saline water.

Numerous researchers had looked into the magnetic water. The examination of magnetic water's effects on the distribution of water uniformity in drip irrigation is not well-trained. Thus, the objectives of this research were to (1) study the effects of magnetic saline water on hydraulic parameters of emitters discharge under different operating pressures; and (2) compare the impacts of using magnetic water along with saline water on clogging characteristics of drip irrigation emitters.

## Materials and methods

These investigations aimed to evaluate using saline and magnetic water to improve drip irrigation management. The present investigation was carried out at the National Irrigation Laboratory of Agricultural Engineering Research Institute (AEnRI), Dokki, Giza, Egypt, to study the effect of salinity and magnetic treatments on hydraulic performance and emitter clogging in surface drip irrigation. The evaluation parameters were to calculate the coefficient of manufacturing variation, emitter discharge coefficient and emission uniformity, in order to establish the emitter´s flow rate sensitivity to salinity and water magnetization.

**Figure 1:**
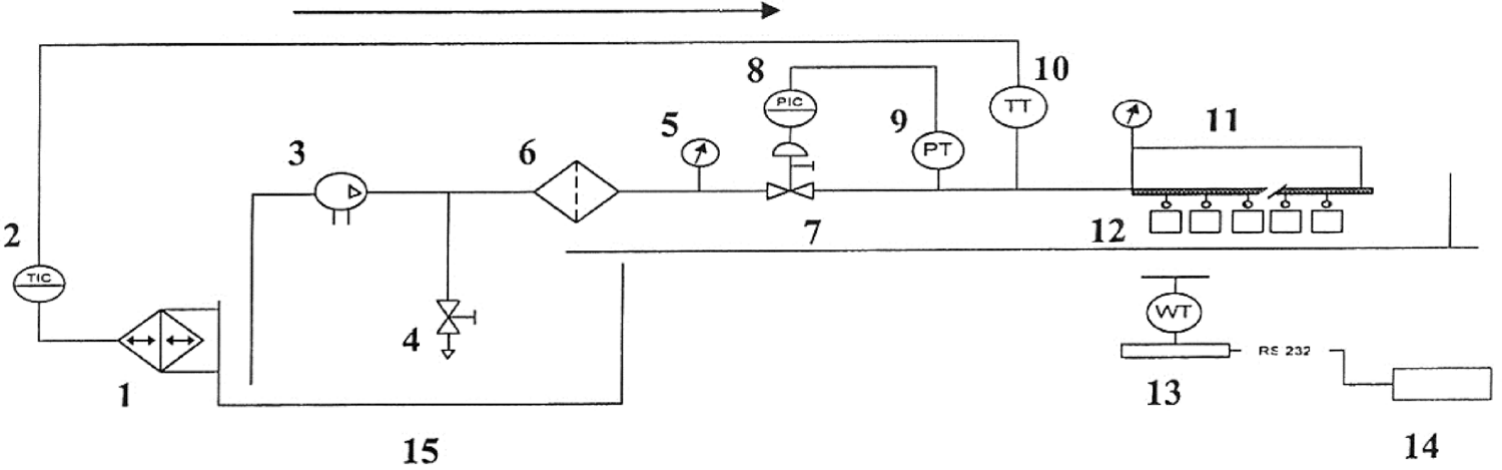
Hydraulic test bench components. 1—Temperature conditioning; 2—Temperature regulator; 3—Multi stage pumping unit; 4—Manual discharge valve; 5—Direct reading pressure gauge; 6—Screen filter; 7—Pressurized air regulating valve; 8—Pressure regulator; 9—Pressure transmitter; 10—Temperature transmitter; 11—Lines of pipes including tested emitters; 12—Water collectors for each emitter in test; 13—Weighing scale; 14—Personal computer; and 15—Water tank.

The drip irrigation systems test facility (Fig. 1) was used to evaluate hydraulic characteristics of emitters.

**Figure 2:**
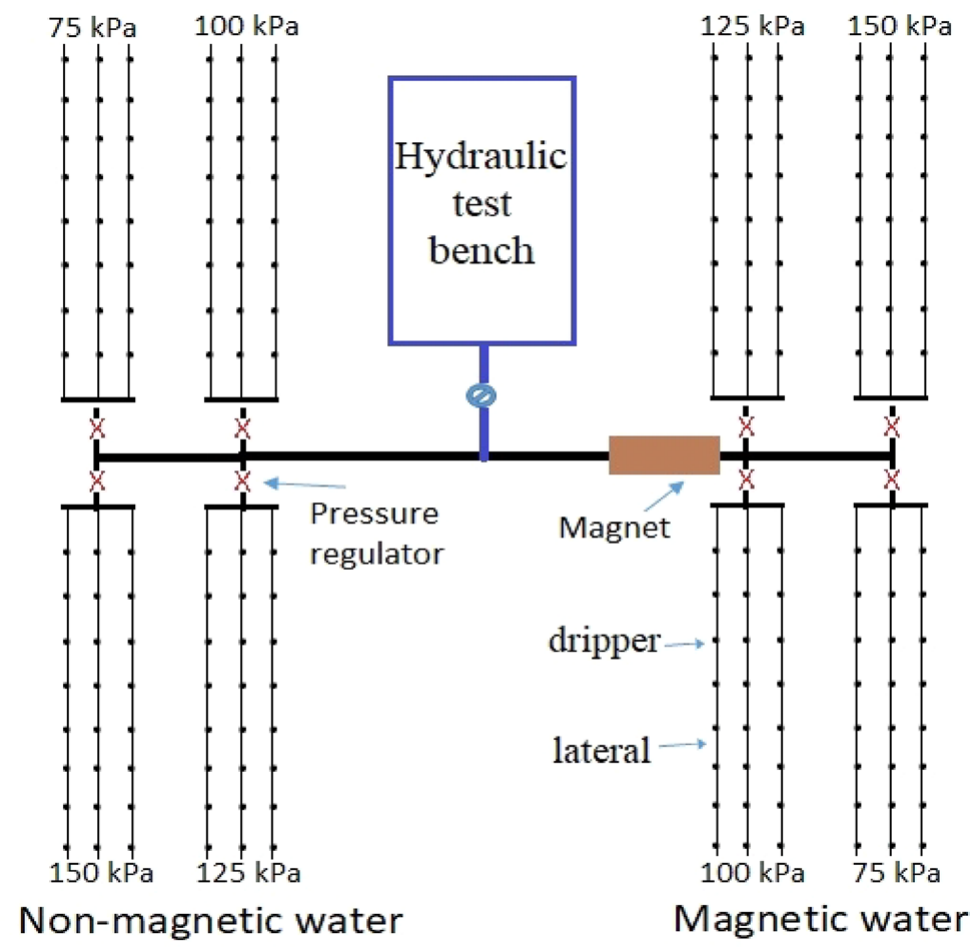
The schematic of experimental system including the magnetic and non-magnetic sub-systems.

**Figure 3:**
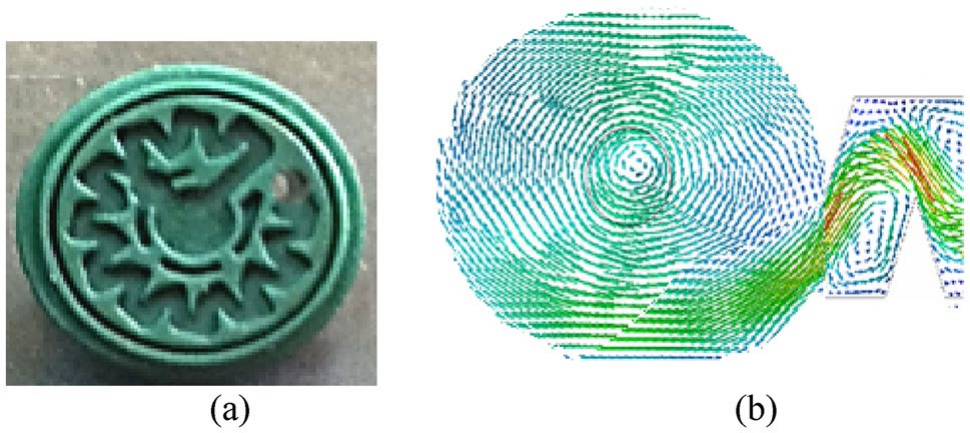
Water path map (**a**) and velocity vortex profile (**b**) in the outlet area of the emitter.

The drip irrigation system consisted of two subunits, one subunit for magnetic water and the other for the non-magnetic water. Each subunit divided to four blocks one for each operating pressure with three drip lines as illustrated in Fig. 2). All pipes used in the system were polyethylene. Laterals with outer diameter of 16 mm, 20 m length and 4 Lh^−1^ on-line emitters were used (On-line turbo emitter types of 4 Lh^−1^ discharge and 5 mm barb outer diameter as shown in Fig. 3). Emitters were set 0.5 m apart.

**Figure 4:**
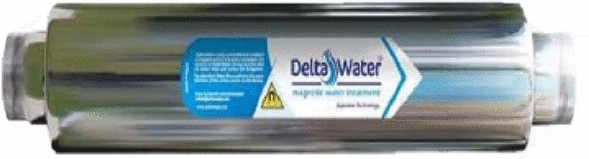
Delta Water Co. magnetic water device.

The magnetization device was supported to the inlet of the magnetic water subunit. The magnetic device is a product of Delta Water Co. for water treatment as shown in Fig. 4. It is constructed from stainless steel material, inner diameter size 2 inches, water flow rate up to 25 m^3^/h, 85 cm length, and 11 kg weight. It is working up to 100 °C temperature, working pressure up to 15 bar, and effective for medium salinity water treatment up to 8000 ppm. With a magnetic capacity of 14,500 Gauss (1.45 Tesla), water passes through the magnetic field and becomes magnetized, which causes some physical changes in the composition and shape of water molecules.

Two main treatments consist of non-magnetic and magnetic irrigation water, and three sub-treatments of three salt concentrations including 219 ppm (tap water as control), 1000 and 2000 ppm were used under different operating pressure (75, 100, 125 and 150 kPa). The salinity levels were prepared by adding Rashidy salt (containing about 99% NaCl, % Na = 31.64% and % Cl = 67.45%) and calcium carbonate to tap water to reach the required salinity that measured by EC-meter.

Irrigation was run 4 h daily for 30 days with totally 120 h for each salinity level^24^. To evaluate the application of treatments on irrigation system hydraulics, hydraulically parameters of the irrigation system were measured^25^. The measured hydraulically parameters were average emitter discharge rate [Eq. (1)], emitter flow variation [Eq. (2)], coefficient of variation [Eq. (3), and emission uniformity [Eq. (4)].

1 $${q}_{a}=\frac{1}{n}{\sum }_{i=1}^{n}{q}_{i}$$

2 $${q}_{var}=100(\frac{{q}_{max}-{q}_{min}}{{q}_{max}})$$

3 $${C}_{v}=100 (\frac{{S}_{q}}{{q}_{a}})$$

4 $${E}_{u}=100 \left(\frac{{q}_{Lq}}{{q}_{a}}\right)$$

where q_a_: average of emitter discharge rates (l/hr), q_var_: emitter flow variation (%), Cv: coefficient of variation (%), Sq: standard deviation of emitter discharge rates (l/h), n: number of tested emitters, qi: individual emitter discharge rates (l/h), Eu: emission uniformity, and q_Lq_: average discharge rate of the lowest one-fourth of the emitter discharge rates (l/h).

Clogging in saline water is closely related to the formation of precipitation and its growth inside emitters. Emitter flow rates were collected at the beginning and the end of the experiment and were measured using the volume method. Based on the measured flow rates, clogging ratio was calculated^26^ as follows:

5 $$CR =100 \left(1-\frac{{q}_{u}}{{q}_{i}}\right)$$

where: CR: emitter clogging ratio, (%), q_u_ = the flow rates at the end (l/hr), and q_i_ = flow rates at the beginning (l/h).

### Ethical approval

We confirm that all methods were carried out in accordance with relevant guidelines in the “Materials and methods” section.

## Results and discussions

At the beginning of the experiment, results show that hydraulic parameters were almost the same for both the magnetized and the non-magnetized water and for all salinity levels since the accumulation of salts has not yet occurred. At the end of working time, the hydraulic parameters were different for the magnetized water under salinity levels compared to the non-magnetized water. Therefore, the focus was on presenting and comparing the results obtained at the end of the experiment to show the effect of magnetization and different salinity levels on the performance of emitters under different operation pressure.

### Emitter flow rate

**Figure 5:**
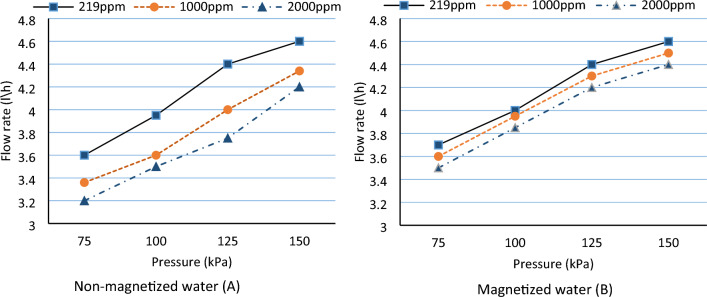
Effect of magnetic and non-magnetic water on flow rate under different salinity and pressure.

The effect of magnetized water on the performance of emitters under various operating pressure and salinities was evaluated according to ASAE, standard^27^. The flow rate using magnetization in the normal conditions (non-saline water) did not change the flow rate of the emitter or affect its behavior. Kiani et al.^28^ found similar results, stating that in non-saline water conditions, did not offer a relatively higher advantage compared to the use of magnetized and non-magnetized water. In contrast, saline water (1000 ppm) the flow rate increased by 10.1, 8.3, 9.25 and 6.9% at 75, 100, 125, and 150 kPa respectively (Fig. 5A) compared to non-magnetized water (Fig. 5B).

In higher salinity (2000 ppm), magnetized treatment increased flow rate by 9.4, 8.6, 11.8 and 7.1% as compared to non-magnetized treatment at the same pressure. The mean flow rate was increased by magnetized water with roughly 2.7% and 5.6 at 1000 ppm and 2000 ppm as compared to non-magnetic treatment, respectively. This results in agreement with^21^ findings, by crossing irrigation water through a magnetic field, the means of the system discharge increased by 4.2%. These results may be due to a decrease in the consolidation degree between water molecules. For these reasons, the viscosity of magnetic water is less than viscosity of non-magnetic water. This result is consistent with^29^.

Increasing the pressure from 75 to 150 kPa, the flow rate increased with the same trend by average of 28.4% and 27.3% for all salinity levels at non-magnetized and magnetized water respectively. The average flow rate as a function of operating pressure was determined for emitters as shown in Table 1 and Fig. 5A and B. All correlation coefficients ranged from 0.93 to 0.94 in non-magnetized water and improved to 0.99 in magnetized water at 1000 and 2000 ppm respectively. Almost all emitters were fully turbulent flow characteristics.

**Table 1:** Regression analysis for flow rate characteristics.

Salinity	Equation	R^2^	SD
Ppm	Non-magnetic water		
219	q = 3.566P^0.181^	0.98	0.45
1000	q = 3.294P^0.182^	0.94	0.43
2000	q = 3.147P^0.184^	0.93	0.42
	Magnetic water		
219	q = 3.663P^0.160^	0.98	0.40
1000	q = 3.578P^0.163^	0.99	0.39
2000	q = 3.485P^0.167^	0.99	0.39

*SD* standard division.

Multi-regression analysis was performed on the data to get equations to describe the relationship between emitter flow rate (q) and operating pressure (P) for three salinity levels and magnetized and non-magnetized water.

### Manufacture’s coefficient of variation (CV %)

**Figure 6:**
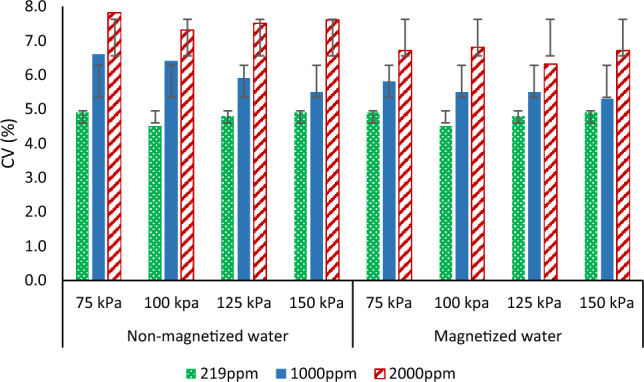
Effect of magnetic on manufacture coefficient of variation (CV %) under different salinity and pressure.

Figure 6 shows the effect of magnetized water on Manufacture’s coefficient of variation. It was noticed that CV values were 4.9, 4.5, 4.8 and 4.9% “Excellent” according to ASAE standard classification that’s at non-salinity has no influence when using magnetized water. However, when salinity increased, CV became 5.5 to 6.6% “average” for 1000 ppm salinity and between 7.3 to 7.8 “marginal” for 2000 ppm salinity. Magnetization improved the performance of the CV of emitter, it reduced about 9.1%, 9.4%, 6.8%, and 5.5% at 75, 100, 125, and 150 kPa respectively, as compared to non-magnetic treatment. At 2000 ppm salinity, a reduction on CV values were noticed and became lower than 7, indicating that the emitter classification improved from marginal to average, which is similar to the results of^21^ who reported that magnetic water decreased CV by 0.98% compared to no-magnetic water.

### Emission uniformity

**Figure 7:**
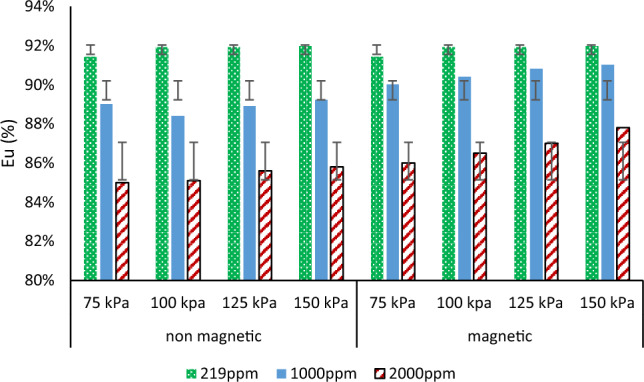
Effect of magnetic and non-magnetic water on emission uniformity, under different salinity and pressure.

In general, magnetization in non-saline water did not affect the emitter’s emission uniformity or behavior, as illustrated in Fig. 7. On the other hand, with increasing water salinity the emission uniformity improved with magnetic treatments.

Emission uniformity increased by roughly 1% with magnetic treatments at different pressure and salinity levels. Eu improved by approximately 0.2, 0.6, 1.1 and 1.8% at 1000 ppm, and by approximately 0.8, 0.9, 1, and 2% at 2000 ppm, as compared to non-magnetic treatments at 75, 100, 125, and 150 kPa respectively. It should be mentioned that the classification went from “good” to “excellent” that in agreement with^30^ who indicated magnetic increased emission uniformity and Christiansen’s Uniformity coefficient.

### Variation of average flow rate (qvar %)

**Figure 8:**
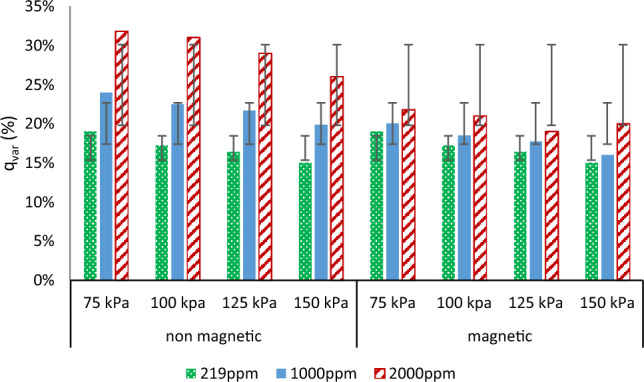
Effect of magnetic and non-magnetic water on variation of average flow rate (qvar) under different salinity and pressure.

Figure 8 shows the effect of magnetized water on variation of average flow rate at different pressure and salinities. Using magnetization under normal conditions (non-saline water) showed no change in the q_var_ of the emitter and had no effect on its behavior. The variation of average flow rate decreased by 20, 21.6, 19.2, and 24.4% for 1000 ppm at 75, 100, 125 and 150 kPa respectively compared to non-magnetic treatments. The average flow rate variation at 2000 ppm was 31.8, 31, 29, and 26% when using non-magnetic water and decreased to 21.8, 21, 19, and 20% with using magnetized water at 75, 100, 125, and150 kPa, respectively. These values show that the use of magnetized water let to improve the flow variation with using low quality water.

### Emitter clogging ratio

The International Organization for Standardization generally states that clogging is identified (as a standard) when the emitter's actual flow rate is less than 75% of the design^8^. Emitter clogging increases with time as the salinity level of irrigation water increases because it affects emitter performance, which correlated with water quality. These results in Fig. 8 reveal that the emitter discharge variations for the magnetic treatment were lower than the non-magnetic which indicates less dripper clogging. Table 2 displayed that magnetized water, salinity and pressure have an effect on the emitter's clogging ratio. Magnetized water had slight effect on clogging in non-saline water at the end of experimental time. At 1000 ppm and 2000 ppm salinity levels, clogging ratio decreased by 1.5, 1.5, 3.7 and 1.2% with 1000 ppm, and 4, 1.1, 3 and 1.6% for 2000 ppm salinity at 75, 100, 125 and 150 kPa, respectively.

**Table 2:** Effect of magnetic and salinity on clogging emitter.

CR (%)	219 ppm				1000 ppm				2000 ppm			
Pressure (kPa)	75	100	125	150	75	100	125	150	75	100	125	150
Non-magnetic	2.3	3	4	4.2	5.3	5.7	9.1	6.7	11.1	9	13.6	8.7
Magnetic	2.2	2.8	3.9	4	3.8	4.2	5.4	5.5	7.1	7.9	10.6	7.1

In comparison to non-magnetized water treatments, the results demonstrate that magnetized water treatment can effectively relieve emitter clogging and delay the occurrence time of clogging. Similar results were obtained by^22,28^.

The possible reasons can be explained as, an decrease in the emitter's q_var_ and increase in Eu indicates that irrigation water is magnetized, which breaks down the original structure of the water molecular groups and transforms them into smaller ones. This increases the activity of the water, improves its solubility, and affects the growth and morphology of scale crystals^12^. It also makes the crystals less adherent to the pipe wall and more easily washed away by the water flow^31^.

## Conclusions

Because of limited water resources, better use of available water resources and low to medium irrigation water salinities for irrigation is important. Using magnetized irrigation water is one technique to improve hydraulic performance for better behavior and plant growth. Overall, the findings demonstrate that drip irrigation performs better with magnetized water than non-magnetized water. When magnetic water was used for irrigation instead of non-magnetic water, the average emitter discharge increased, indicating less emitter clogging and high distribution uniformity.

The findings showed that the salinity of the water had an impact on the hydraulic parameters. The average emitter discharge, the coefficient of variation, the emission uniformity, and the variations in the emitter discharge were all significantly impacted by the magnetic water. Furthermore, higher emitter performance had better efficiency for reducing emitter clogging. According to this study, using magnetized water treatments to treat water can be an efficient and eco-friendly way to address emitter clogging issues in drip irrigation systems that occur when saline water is carried out.

## Data Availability

All data generated or analyzed during this study are included in this published article. Appropriate permissions and/or licenses for collection of plant or seed specimens is not relevant.
